# Direct label-free measurement of the distribution of small molecular weight compound inside thick biological tissue using coherent Raman microspectroscopy

**DOI:** 10.1038/srep13868

**Published:** 2015-09-10

**Authors:** Masahiko Kawagishi, Yuki Obara, Takayuki Suzuki, Masumi Hayashi, Kazuhiko Misawa, Sumio Terada

**Affiliations:** 1 Department of Neuroanatomy and Cellular Neurobiology, & Center for Brain Integration Research, Graduate School of Medical and Dental Sciences, Tokyo Medical and Dental University (TMDU), Tokyo, Japan; 2 Department of Applied Physics, Tokyo University of Agriculture and Technology (TUAT), Koganei, Japan; 3 Wired Co., Ltd., Komae, Japan; 4 Interdisciplinary Research Unit in Photon-nano Science, Tokyo University of Agriculture and Technology (TUAT), Koganei, Japan; 5 Development of Advanced Measurement and Analysis Systems (SENTAN), Japan Science and Technology Agency (JST), Tokyo, Japan

## Abstract

Distributions of small molecular weight (less than 300 Da) compounds inside biological tissue have been obscure because of the lack of appropriate methods to measure them. Although fluorescence techniques are widely used to characterise the localisation of large biomolecules, they cannot be easily applied to the cases with small molecule compounds. We used CARS spectroscopy to detect and identify a label-free small molecule compound. To facilitate detection in aqueous environment, we utilised time-resolved and phase-sensitive techniques to reduce non-resonant background generated from water. We applied this technique to detect small molecular weight compound, taurine, inside mouse cornea tissue immersed in taurine solution as an initial model experiment. We detected a Raman peak of taurine near wavenumber 1033 cm^−1^ inside cornea and successfully characterised its depth profile in the tissue. Our CARS spectra measurement can be a promising method to measure and visualise the distribution of small bio-related compounds in biological background without using any labeling, paving the way for new cell biological analysis in various disciplines.

Advancements in fluorescent dyes and proteins revolutionised the research about the behaviour and distribution of biological macromolecules, such as proteins and nucleic acids^1^,^2^. Fluorescent probes with specialised optical properties are combined with new microscopic technologies and are used to visualise biological molecules at superresolution^3^. These light-emitting probes enabled a high signal-to-noise ratio imaging of very small target objects. This underscores the strength of visualisation tools in biological research.

But in contrast to the advancements in the study of large biomolecules, our knowledge about the distributions of small molecular weight (less than 300 Da) organic compounds inside biological tissue is still very limited. This is because of the lack of appropriate methods to measure them. Fluorescent labels are relatively large compared to the target compounds and can interfere with their chemical properties. So fluorescence methods could not be easily applied to the cases with small molecule compounds. Thus, a visualisation technique that works without labeling is required. Infrared spectroscopy is used to get label-free information about small molecules. It uses the spectral pattern of infrared absorption that is characteristic to each compound, to differentiate target chemical species and to perform spatial imaging^4^. Fourier Transform Infrared (FT-IR) spectroscopic imaging has been used for many applications, such as probing the composition of lipid, DNA, protein, and other components in cells or tissues^5^, and, combined with statistical classification, has been used to probe and classify microorganisms and cell types^6^,^7^. But because of infrared absorption by water, infrared spectroscopy can only be performed on processed and dried biological samples. The long wavelength of infrared ray also limits the microscopic resolution. Many studies used the peaks in the mid-infrared range of about wavenumber 4000–1500 cm^−1^, the functional group region that includes many stretching vibrations of covalent diatomic units, to differentiate molecular composition of the object, typically lipid content. Because of the limited variation of chemical bonds in biomolecules, gross categorisation, such as lipids and DNAs, was possible, but finer identification on chemical species was not easy in biological samples.

Raman spectroscopy probes molecular vibrations of energy ranges similar to those probed in infrared spectroscopy. It is less affected by water, but spontaneous Raman scattering is typically weak. It has been used for imaging cell chemical composition^8^,^9^,^10^,^11^ and for label-free detection of histological structures^12^,^13^. Coherent anti-Stokes Raman scattering (CARS) is a third-order nonlinear optical process to generate a coherent Raman signal that is enhanced by resonance^14^,^15^,^16^,^17^,^18^,^19^. Multiplex CARS uses pulses with broad spectral width and allows for simultaneous detection of peaks in a wide range of Raman shifts^20^,^21^,^22^,^23^. CARS generated signals have a component that depends on the vibrational mode of a molecule and a component that is purely electronic. These components are referred as resonant and non-resonant, respectively. Resonant signals probe Raman active modes and are of interest, but non-resonant component causes a significant background. Water is a solvent that generates strong non-resonant background (NRB), so a way to extract the weak resonant signal out of strong NRB is essential for observation in biological samples. Several methods have been proposed to circumvent NRB, including time-resolved CARS^24^,^25^, heterodyne interferometric CARS^26^,^27^,^28^,^29^,^30^,^31^, phase-retrieval CARS^32^,^33^. CARS imaging has been used for label-free cell typing and histology^34^,^35^, and for probing lipid compositions^36^.

We have explored the application of CARS spectroscopy to detect and visualise the distribution of small molecule compounds. We used a single-beam heterodyne CARS with shaped broadband pulses and detected inhalational anaesthetic sevoflurane injected into squid axoplasm^37^,^38^. However, fluctuations in the spectral profile and intensity of NRB limited the signal-to-noise ratio of the resonant signal, and thus limited the sensitivity of the measurement. We recently combined phase-sensitive CARS with time-resolved technique^39^,^40^. This effectively removed NRB generated from water. In the present study, we applied our technique to detect a small molecule compound in aqueous biological environment. We successfully identified label-free taurine inside cornea sample and measured its depth profile.

## Results

### Phase-sensitive and time-resolved detection

**Figure 1:**
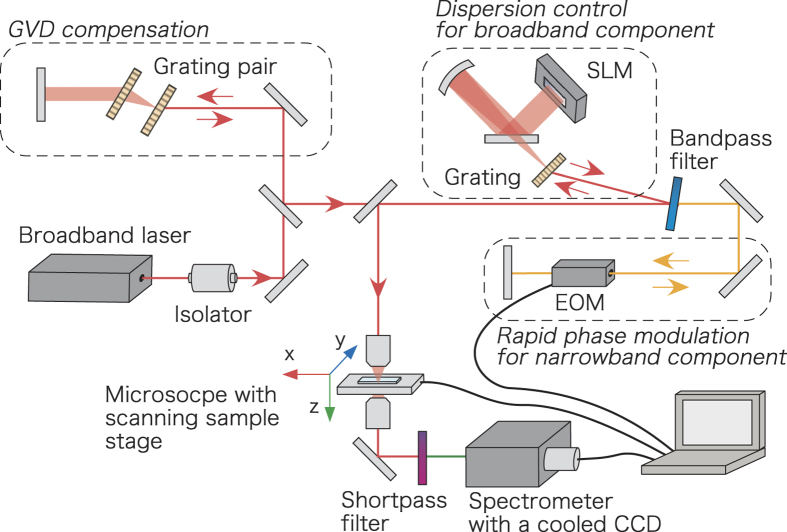
Experiment setup. Output pulse is divided into two pulses: One is the broadband pulse (red line) and the other is narrowband pulse (yellow line). These act as pump and Stokes pulses, and as probe pulse, respectively. The narrowband pulse was passed through and electro-optic modulator, recombined with the broadband pulse, and focused by an object lens onto a sample on the 3D piezoelectric stage. The incident pulses were cut off using short-pass filter, and resonant CARS signals with shorter wavelength were introduced into a spectrometer with a coolant CCD camera.

We recently constructed a CARS microscopy system utilising time-resolved technique and phase-sensitive detection^39^. A schematic representation of our CARS setup is shown in Fig. 1. Laser pulses were divided by a band-pass filter into broadband and narrowband pulses. The narrowband pulse was given a temporal delay, phase-modulated using an electro-optic modulator, and then was used as the probe pulse. The broadband pulse was used for pump and Stokes pulse. Temporal delay of probe pulse of 0.5–1.8 ps effectively removed NRB from water. The broadband and narrowband pulses were recombined and focused on the object. We modulated the phase of the probe pulse and measured the interference spectra between resonant CARS signal and NRB. Then, the resonant CARS spectrum was extracted by taking the phase-synchronised component. The advantage of this method is that the intensity of the resonant signal can be evaluated directly without any assumptions because NRB can be effectively removed experimentally. This method allows for quantitative evaluation of the molecular concentration from the intensity of the resonant signal.

### Resonant CARS measurement of water and taurine solution

**Figure 2:**
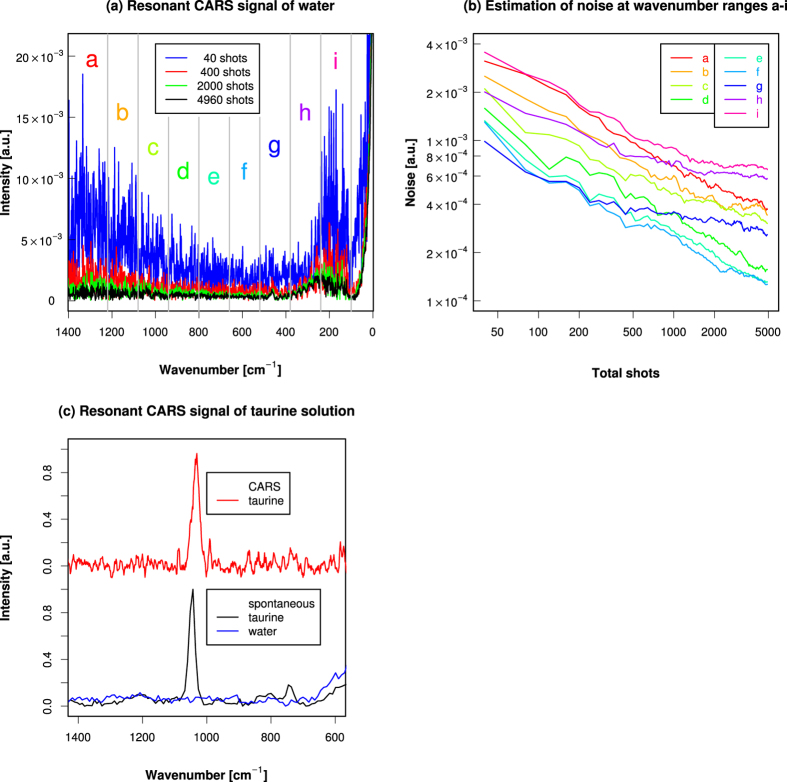
(**a**) Resonant CARS signals obtained from distilled water. The steep slope of incident probe pulse that passed through the short-pass filter was found around wavenumber of 0 cm^−1^, and its interference was seen at wavenumber <500 cm^−1^. Water showed no resonant CARS peaks in this region, confirming a good removal of NRB by our time-resolved detection. The amounts of detection noises differed at various wavenumber ranges. These differences were thought to be influenced by the size of NRB that worked as a local oscillator in phase-sensitive heterodyne detection. Note the large detection noises in the acquisition with a small number of shots that could be reduced by increasing the number of shots. The detection wavenumber range was divided into 9 regions a-i as shown. (**b**) Estimation of detection noise at wavenumber ranges a-i. The standard errors of the mean of the accumulated signals were plotted against the number of shots. For wavenumber >500 cm^−1^ (ranges a-f), the estimated noises decreased as the number of shots increased, with the inverse square root. For wavenumber <500 cm^−1^ (ranges g-i), the noises initially decreased with the inverse square root of shots, but as the number of shots increased, the decreases became slower. This was thought to be due to the strong noise from the interference by the incident probe pulse that leaked in these regions through the short-cut filter. (**c**) Normalised spectra from 0.4 M taurine aqueous solution using resonant CARS (top) and spontaneous Raman (botton). The peak wavenumber was 1033 cm^−1^ for CARS and 1043 cm^−1^ for spontaneous Raman. and difference was smaller than the spectral resolution of 13 cm^−1^ for CARS and 14.5 cm^−1^ for spontaneous Raman, respectively. The spontaneous Raman spectra of water is also shown (blue).

We applied the technique described above to test how low non-resonant CARS signal from water can be suppressed (Fig. 2a). We measured CARS spectra of the range <1500 cm^−1^. This spectral range is the fingerprint region that includes peaks of bending and other vibrations, that are intrinsic to and characteristic of chemical species. Water is known to be the main source of NRB, but we detected no resonant CARS signal peaks from water^39^. This confirmed the effective removal of NRB by our time-resolved detection scheme. However, we found some detection noises that overlaid the signals. These noises came from the fluctuation of the intensity of the pump and probe laser, small vibrations of our optic setup, and the noise from CCD detection. We estimated the size of the noise by taking the standard error of the mean of the accumulated shots of spectral data (Fig. 2b). The noise was stochastic and decreased as the number of shots increased, with the inverse square root. At around 1033 cm^−1^, the detection noise was estimated to be ±2 × 10^−4^ a.u.

We then used the technique to detect small molecular weight compound in aqueous environment. As an example, we tested taurine (2-aminoethanesulfonic acid, H_2_N-CH_2_-CH_2_-SO_3_H). Taurine is a naturally occurring *β*-sulfonated organic acid and is one of the most abundant free organic acids in animal body. It is widely present in various tissues, with high concentrations found in platelets, electrically excitable tissues, and secretory structures^41^. It is reported to have various physiological functions and roles including conjugation of bile acids, antioxidation, anti-inflammation, neuromodulation^42^,^43^,^44^,^45^,^46^. But its fine distribution in cells and tissues is not well known. Several methods such as chromatography and mass spectrometry have been used to detect taurine in low concentration^47^,^48^,^49^,^50^,^51^, but it was not easy to get spatial concentration profiles. Infrared spectroscopy can be used to get spatial information, but it required processed and dried sample^52^. Taurine is soluble in water and gives strong CARS signal, so CARS microscopy can be a good sensitive method to get the concentration of taurine in wet sample with local spatial information. We measured CARS spectra in the fingerprint region of 0.4 M taurine aqueous solution. A major peak of taurine was observed near 1033 cm^−1^ (Fig. 2c). This peak was attributable to the sulfonic acid group of taurine^53^,^54^. The spontaneous Raman spectra of taurine in aqueous solution showed a strong peak at 1043 cm^−1^ (Fig. 2c). The difference is within the spectral resolution of the measurements. The spectral width was about 13 cm^−1^ which reflected the width of narrowband probe pulse. The peak intensity of the 1033 cm^−1^ peak of taurine was about 1 × 10^−2^ a.u., so the signal-to-noise ratio was estimated to be 30–40.

### CARS measurement of taurine and its depth profile

**Figure 3:**
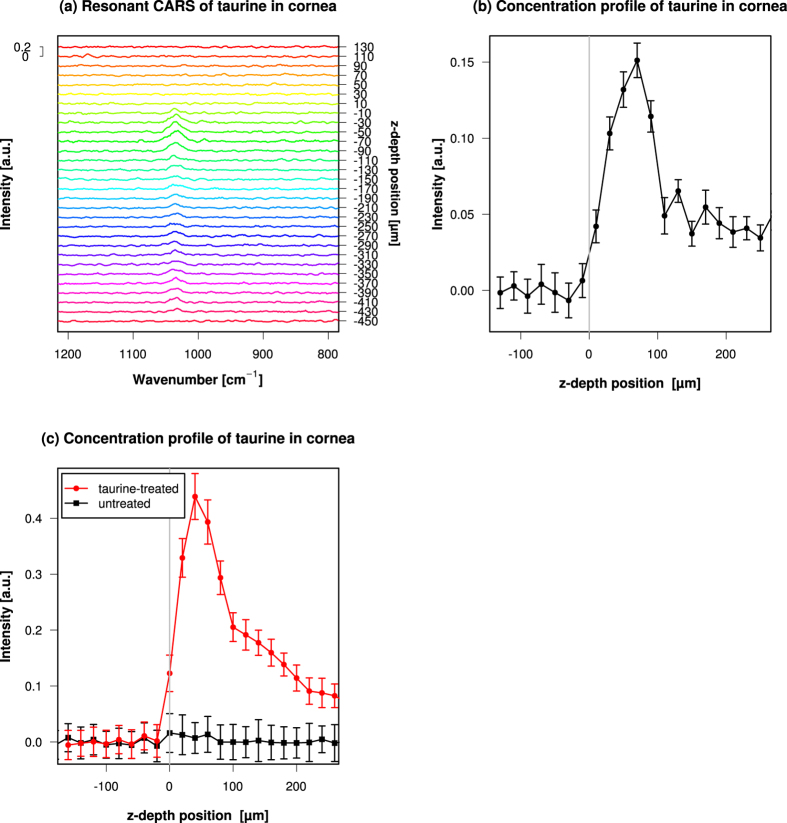
Spectra and concentration profile of CARS measurements obtained from mouse cornea sample immersed in taurine aqueous solution. (**a**) z-Axial scan of the cornea tissue. The position of the focus of incident pulses was varied along the stage z-axis inside the cornea sample. z-Depth position of 0 *μ*m was set at the epithelial surface of the cornea. The plus direction is toward the endothelial side of the cornea, and the minus direction is toward the silicon oil around the cornea sample. 1000 shots of spectral data were acquired at each z-Depth position to get the resonant CARS spectra. Resonant CARS signals around wavenumber 1033 cm^−1^ of taurine were observed. (**b**) Relative concentration profile of taurine in cornea tissue immersed in taurine aqueous solution. The intensity of wavenumber 1033 cm^−1^ peak of resonant CARS signal was integrated over the range of the peak. The error bars were estimate as described in Methods. Note that the size of the noises is relatively stable over the range of z-depth positions. (**c**) Relative concentration profile of taurine from a cornea sample obtained from another measument session. Profiles of taurine from corneas treated with or not treated with taurine solution are shown. The error bars were estimate as described in Methods.

As an initial model of CARS measurement in an aqueous biological sample, we measured taurine inside mouse cornea tissue. We chose cornea because it is transparent and is suited for optical measurement. We fixed mouse eye, immersed it in taurine solution, dissected the cornea, washed it briefly, and placed it in a drop of silicon oil on a glass slide. We placed the spherical cap-shaped cornea sample on the microscope stage with the base at the bottom and the convex surface facing the top, and measured the 1033 cm^−1^ peak of taurine inside the tissue. The axial position of the sample was moved with a 3D piezoelectric stage, and we scanned through the depth of the tissue. We successfully acquired resonant CARS signals (Fig. 3a). We integrated their intensities across the tissue to get the depth profile of taurine (Fig. 3b,c). This takes advantage of the high spatial resolution of CARS due to its property as a three-photon nonlinear process. This direct measurement of the concentration profile at this resolution in z-axial direction was not possible with conventional infrared microscopy. We did not observed any resonant CARS signals around 1033 cm^−1^ from cornea not treated with taurine (Fig. 3c), so the 1033 cm^−1^ signal is attributable not to endogenous material of cornea but to externally applied taurine. In the resonant CARS profile shown in Fig. 3a,b, we could see strong signals of taurine from depth positions 10 *μ*m to 90 *μ*m with a peak at 70 *μ*m. The thickness of the central part of mouse cornea is 120–160 *μ*m, and thus we detected the taurine resonant CARS signal inside cornea tissue. We observed weaker CARS signals at depth positions >160 *μ*m, way beyond the thickness of cornea. This reflects the small amount of taurine that leaked out of the tissue into the buffer that remained on the concave surface of cornea. We also observed cases where the peak of the depth profile of taurine was located near the epithelial surface of cornea. In the resonant CARS profile shown in Fig. 3c, we could see strong signals of taurine from depth positions 0 *μ*m to 100 *μ*m with a peak at 40 *μ*m. This was not the middle of the 140 *μ*m thickness of cornea but was skewed toward the epithelial side.

**Figure 4:**
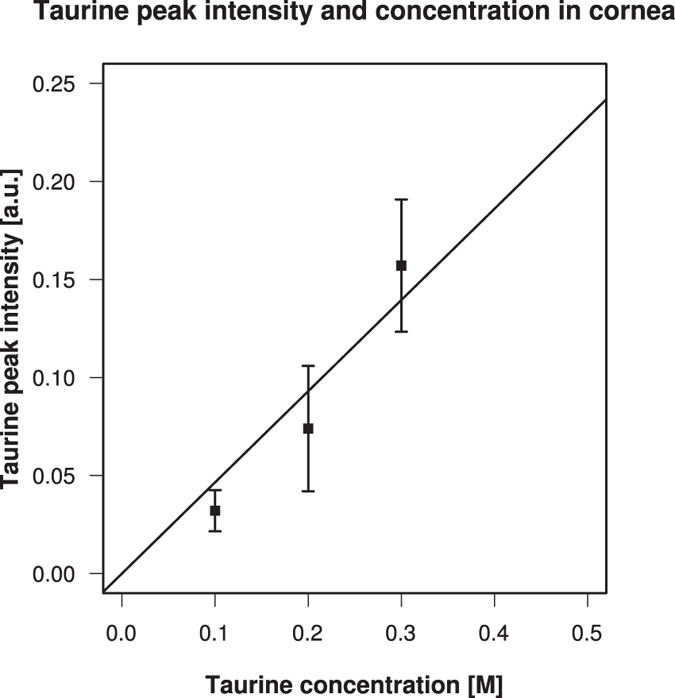
Relationship between taurine resonant CARS peak intensity and concentration. Cornea was immersed in taurine aqueous solutions of 0.1, 0,2, and 0.3 M, and the concentration profiles were measured using resonant CARS signals (Supplementary Figure S1). Peak intensities of taurine were plotted against the concentration of the solutions. A linear relationship *y* = 0.4651*x* was observed. The error bars were estimate as described in Methods.

We then tested the ability of our CARS setup to quantify the concentration inside biological tissue. Cornea tissue fixed in formaldehyde is opaque, and shows a lot of scattering of light. We tested mouse corneas immersed in taurine aqueous solutions of various concentrations, and found a linear relationship between the peak intensity of CARS signals and the concentration of the solutions (Fig. 4). These data show that this technique can give quantitative data proportional to the concentration in biological tissue if the tissue context is stable and does not change. Thus, this technique is suitable for quantifying the local relative concentration profiles.

**Figure 5:**
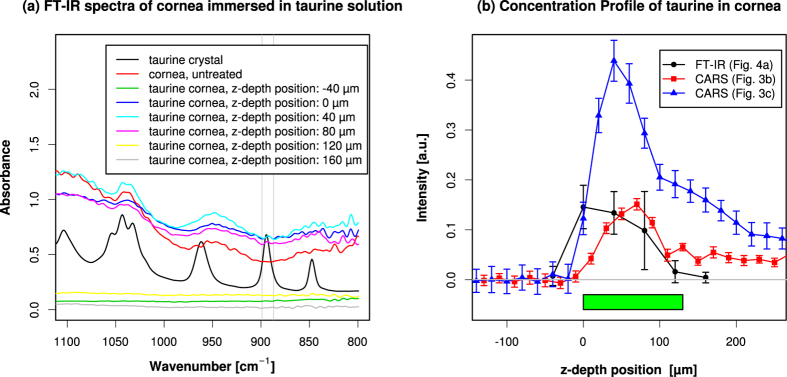
The depth profile of taurine measured using resonant CARS and FT-IR. (**a**) Typical raw spectra of cornea tissue obtained using FT-IR microscope. Cornea immersed in taurine was frozen, sectioned, dried, and observed using FT-IR microscope. Several regions of interest of the size 40 *μ*m × 40 *μ*m were set across the thickness of the cornea, and the spectra were measured. The z-depth positon of –40 *μ*m corresponded to the area outside of cornea adjacent to the epithelial surface. The thickness of the cornea was about 140 *μ*m, and the z-depth positon of 120 *μ*m corresponded to the area where the endothelial edge was in the middle. The FT-IR spectra of taurine crystal and non-taurine-treated cornea are also shown. The peak of 893–894 cm^−1^ was used for integration and quantification, and the ±6 cm^−1^ range is indicated by thin gray lines. A broader wavenumber range is shown in Supplementary Figure S2. (**b**) Comparison of concentration profiles of taurine in cornea tissue obtained using FT-IR (black dots) and resonant CARS (red square and blue triangle). Position of zero was set at the epithelial surface of the cornea. Error bar: estimate of noise as described in Methods for resonant CARS, standard error of the mean (n = 6) for FT-IR. The light green bar above the horizontal axis shows the approximate thickness of the cornea sample.

The asymmetric distribution of taurine, skewed toward the epithelial side, in fixed cornea was unexpected. To validate the depth profile measurement of taurine using resonant CARS, we compared it to the result obtained by another method. We used FT-IR spectroscopy to detect taurine^52^. We froze the taurine-immersed cornea sample, sectioned and dried it, and measured the 893–894 cm^−1^ peak of taurine^55^ using FT-IR microscope (Fig. 5). We could detect the taurine signal from depth positions from the full thickness of the cornea, and we found a slight increase of the concentration toward the epithelial side, but the diffrence was small, and limited resolution of FT-IR did not allow further quantification. In contrast, resonant CARS measurement showed higher signal-to-noise ratio and higher spatial resolution. This also validated the reliability of CARS microscopy as a new method to quantify the depth profile of taurine. FT-IR microscope required sectioning of the sample because of the low spatial resolution limited by long wavelength. FT-IR microscope also required drying of the sample because of infrared absorption by water. However, CARS microscope could detect taurine in wet tissue block. This takes advantage of the higher spatial resolution and effective NRB removal of CARS in our setup. This underscores the strength of CARS microscopy for characterisation of small molecule compound in wet biological tissues.

## Discussion

We detected CARS peak of taurine near 1033 cm^−1^ inside cornea, and characterised its depth profile in the tissue. This measurement utilised the Raman spectra specific to the target compound and required no labeling. Our CARS setup could measure the intensity of resonant CARS signal without numerical reduction methods^33^,^36^, and thus allowed for quantitative evaluation of the local relative concentration profiles (Fig. 4). This feature is useful in cases of measuring other small molecular weight molecules where labeling interferes with the chemical properties of the target.

We sometimes observed an asymmetric peak in the taurine depth profile skewed toward the epithelial side (Fig. 3c). We prepared the sample simply by applying taurine from outside to fixed cornea sample, and then washing it. It was very interesting to observe a non-uniform distribution in the concentration of a soluble small molecule compound in a fixed sample. We think this reflected the difference in permeability between the epithelium and endothelium of the cornea. Stratified corneal epithelium has tight junctions (TJs) at apical cell-cell contacts^56^ and they form a barrier that separates the eye from the outside. On the other hand, TJs of corneal endothelium have focal missing parts and are known to be leaky^57^,^58^. This is thought to be a part of the structural basis of the Pump-Leak hypothesis that controls the physiological hydration and fluid turnover of corneal stroma^59^,^60^. Corneal stroma is an avascular tissue, and the passage of fluid and nutrition into and out of stroma is controlled by the endothelium. The leaky paracellular entry of fluid into the stroma is thought to be counterbalanced by the active pump of endothelial cells to keep the physiological hydration and to prevent edema. In our fixed cornea sample, this leaky endothelium caused a higher permeability of small molecule compounds. When our cornea sample was immersed in taurine, more taurine moved into the stroma through the endothelial side than through the epithelial side, and when the sample was washed, more taurine moved out through the endothelial side and thus created the asymmetric distribution profile. It was interesting that this difference could be visualised using CARS microscopy.

We used CARS and FT-IR to obtain the depth profile of taurine. The spatial resolution of FT-IR microscope is limited by the wavelength of infrared light. In z-axial direction the resolution can be several tens of micrometers for a light of 1033 cm^−1^. But CARS is a third-order optical process and has higher resolution both in xy and z directions. So we could measure the z-direction profile without the need for sectioning the sample. Our time-resolved method could effectively remove NRB from water. These features are useful for measuring the fine spatial profile in a wet biological sample with significant volume. FT-IR measures the absorption of infrared light by the target, which limits its dynamic range. On the other hand, CARS measures the emission of CARS ray from the target. CARS has a potential for increasing the signal-to-noise ratio by using a longer acquisition time (Fig. 2a,b).

Thus spectra measurement and microscopy using our phase-sensitive CARS with time-resolved technique can be a promising method to characterise and visualise the distribution of small bio-related compounds in wet biological background without using any labeling, paving the way for new cell biological analysis in various disciplines.

## Methods

### CARS optical setup

A schematic representation of our CARS setup is shown in Fig. 1. We used a single femtosecond laser oscillator (Vitara-T, Coherent) as a broadband laser source. The center wavelength, bandwidth, and repetition rate are 800 nm, ~100 nm, and 80 MHz, respectively. The pulse was then divided into two pulses by a band-pass filter: One was a broadband pulse which acts as pump and Stokes pulses. The other was a narrowband pulse with a spectral width of 4 nm, which had a role of probe pulse. The broadband pulse was pre-compensated for the spectral dispersion from the objective lens by using the transmission grating and a reflective type liquid crystal spatial light modulator (LCOS-SLM X10468-02, Hamamatsu Photonics). The narrowband pulse was passed through an electro-optic modulator (EOM) for rapid phase modulation, and then recombined with the broadband pulse. The recombined pulses were focused with an objective lens (M Plan Apo NIR HR 100×, Mitsutoyo) to a diameter of 1.7 *μ*m. The energy of the combined pulses was less than 1 nJ at the sample.

Forward-scattered CARS signals were collinear with the incident pulses, and so the signals were collimated after the sample. The signal was passed through a short-pass filter to cut off the incident pulses spectrally, and introduced into a spectrometer with a cooled CCD camera (Oriel MS260i + Andor DV420). A set of four shots of spectral data were acquired with varying phase modulation of probe pulse and were accumulated from 250 sets (1000 shots) to 2500 sets (100000 shots).

The resonant CARS signal was obtained as an interference pattern superimposed on intense and broad non-resonant background which worked as a local oscillator. Since the interference term is phase-synchronised with the probe modulation, only the modulated resonant signal was extracted by the phase-sensitive heterodyne detection. To improved the signal-to-noise ratio, we smoothed the data by moving average method with the window width of 13 cm^−1^ which corresponds to the spectral resolution of our setup determined by the width of narrowband probe pulse. The intensity of 1033 cm^−1^ peak was estimated by integrating the resonant CARS signal over the peak range and dividing by the number of CCD pixels in the peak range. For each z-depth position, the spectral signals from 825–976 cm^−1^ where there is no resonant CARS peaks were used to calculate the standard error as an estimate of noise of a single spectral data point. When the CARS signals were integrated over the range of the peak to get the total intensity of the peak, noise of one spectral data point was multplied by the square root of the number of spectral data points in a peak to obtain an estimate of the noise of total intensity of the peak. This estimate of noise was used as the error bar of concentration profiles.

### Preparation of mouse cornea

Adult mice (BALB/c, 7–9 weeks old) were euthanatised. The eyes were enucleated and fixed with 4% formaldehyde in phosphate-buffered saline (137 mM NaCl, 2.7 mM KCl, 8.1 mM Na_2_HPO_4_, 1.1 mM KH_2_PO_4_). The fixed eyes were immersed in 0, 0.1, 0.2, 0.3, 0.4 M or saturated taurine aqueous solutions overnight. Then the cornea was dissected, and the central part was trimmed into a convex square of about 3 mm. The trimmed tissue was washed briefly with phosphate-buffered saline, placed in a drop of silicon oil on a glass slide with a concave cavity, and sealed by a coverslip with manicure. The sample was set on a three-dimensional piezoelectric stage under CARS microscope. All animal experiments were carried out in accordance with the rules set by the Institutional Animal Care and Use Committee of TMDU. The experiments were approved by the Institutional Animal Care and Use Committee of TMDU (Nos 0140220A, 0150142A, 0160012A).

### Fourier transform infrared microscopy

Mouse cornea sample was frozen and sectioned using cryostat microtome (Cryostat 2800 Frigocut-E, Reichert-Jung). The slicing plane was set parallel to the axis of the eye and perpendicular to the base of the spherical cap shape of cornea. The thickness of each slice was 30 *μ*m. The slices were recovered on gold-coated glass slides, dried, and observed using infrared microscope (IRT-5200, JASCO) and Fourier transform infrared spectrometer (FT/IR-4700 type A, JASCO). Several regions of interest of the size 40 *μ*m × 40 *μ*m were set across the cornea sample, and the spectra were measured. We tried smaller regions of interest to get higher spatial resolution, but they degraded the quality of the spectra and made the quantification unstable. We used the absorption of the peak at 893–894 cm^−1^ for quantification because this peak was located in a wavenumber where the absorption was low and relatively flat in untreated cornea samples (Fig. 5a).

## Additional Information

**How to cite this article**: Kawagishi, M. *et al.* Direct label-free measurement of the distribution of small molecular weight compound inside thick biological tissue using coherent Raman microspectroscopy. *Sci. Rep.* **5**, 13868; doi: 10.1038/srep13868 (2015).

## Supplementary Material

Supplementary Information
