# Supplementary material for: Direct label-free measurement of the distribution of small molecular weight compound inside thick biological tissue using coherent Raman microspectroscopy
Source: Sci Rep. 2015 Sep 10;5:13868. doi: 10.1038/srep13868 (PMC4564726; doi:10.1038/srep13868)

Title

Direct label-free measurement of the distribution of small molecular weight compound inside thick biological tissue using coherent Raman microspectroscopy

Authors

- Masahiko Kawagishi[1]
- Yuki Obara[2]
- Takayuki Suzuki[2]
- Masumi Hayashi[3,5]
- Kazuhiko Misawa[2,4,5]
- Sumio Terada[1,5,*]

Affiliations

- [1]Department of Neuroanatomy and Cellular Neurobiology, & Center for Brain Integration Research, Graduate School of Medical and Dental Sciences, Tokyo Medical and Dental University (TMDU), Tokyo, Japan
- [2]Department of Applied Physics, Tokyo University of Agriculture and Technology (TUAT), Koganei, Japan
- [3]Wired Co., Ltd., Komae, Japan.
- [4]Interdisciplinary Research Unit in Photon-nano Science, Tokyo University of Agriculture and Technology (TUAT), Koganei, Japan
- [5]Development of Advanced Measurement and Analysis Systems (SENTAN), Japan Science and Technology Agency (JST), Tokyo, Japan
- [*]Corresponding author

Supplementary Figure S1.

Concentration profiles of taurine in cornea immersed in taurine aqueous solutions of various concentration. Corneas were immersed in taurine aqueous solutions of (a) 0.3 M, (b) 0.2 M, and (c) 0.1 M, and the concentration profiles were measured using resonant CARS signals. 1000 (ab) or 10000 (c) shots of spectral data were acquired at each z-Depth position to get the resonant CARS spectra. Depth profiles of resonant CARS signals of silicon oil are also shown the mark the boundary of cornea tissues. These data were used to calculate the peak intensities of taurine used in Figure 4.

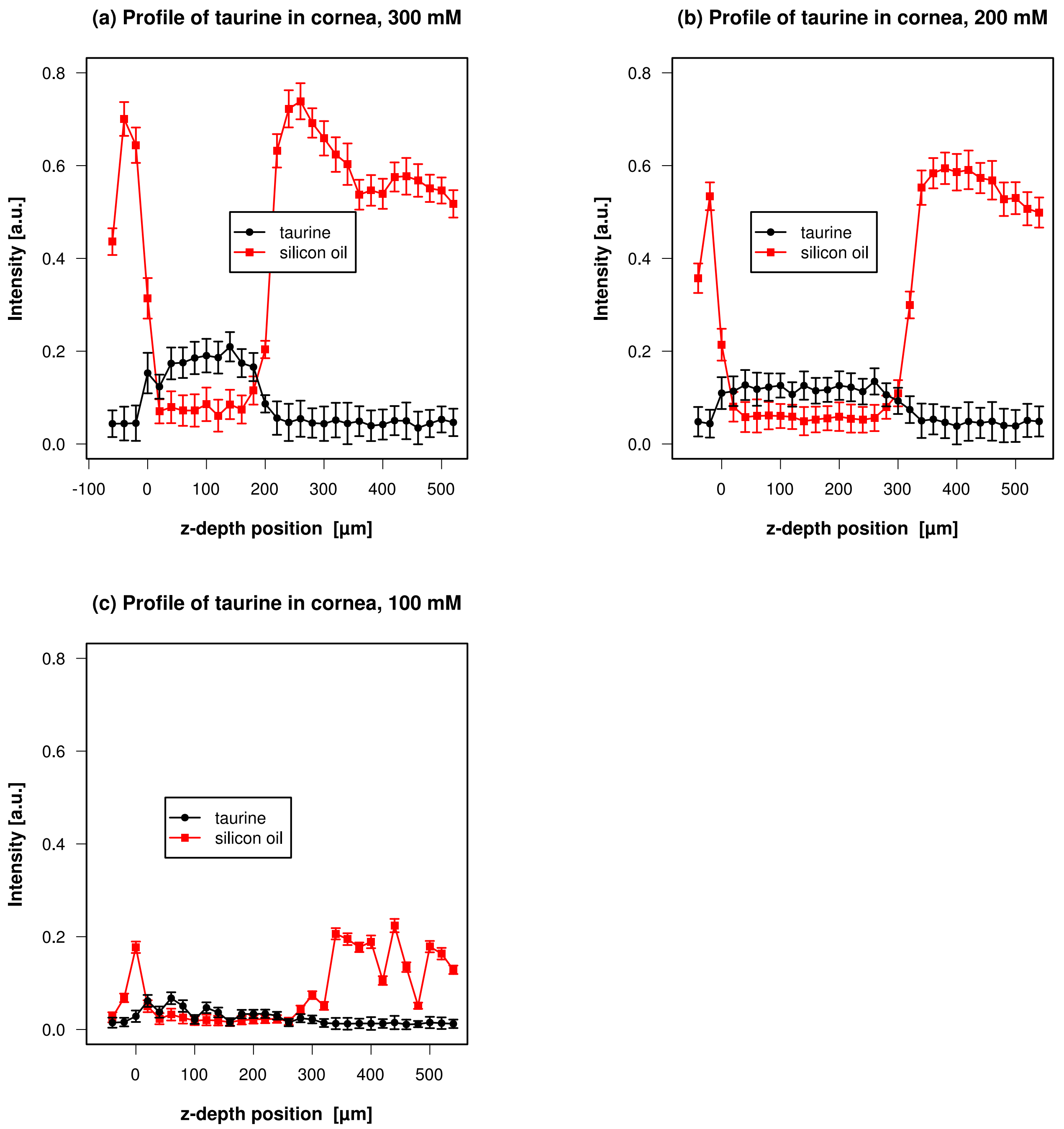

Supplementary Figure S2.

Typcal raw spectra obtained from cornea tissues using FT-IR microscope. These are the same datasets as those used shown in Figure 5a, but broader wavenumber range is shown here.

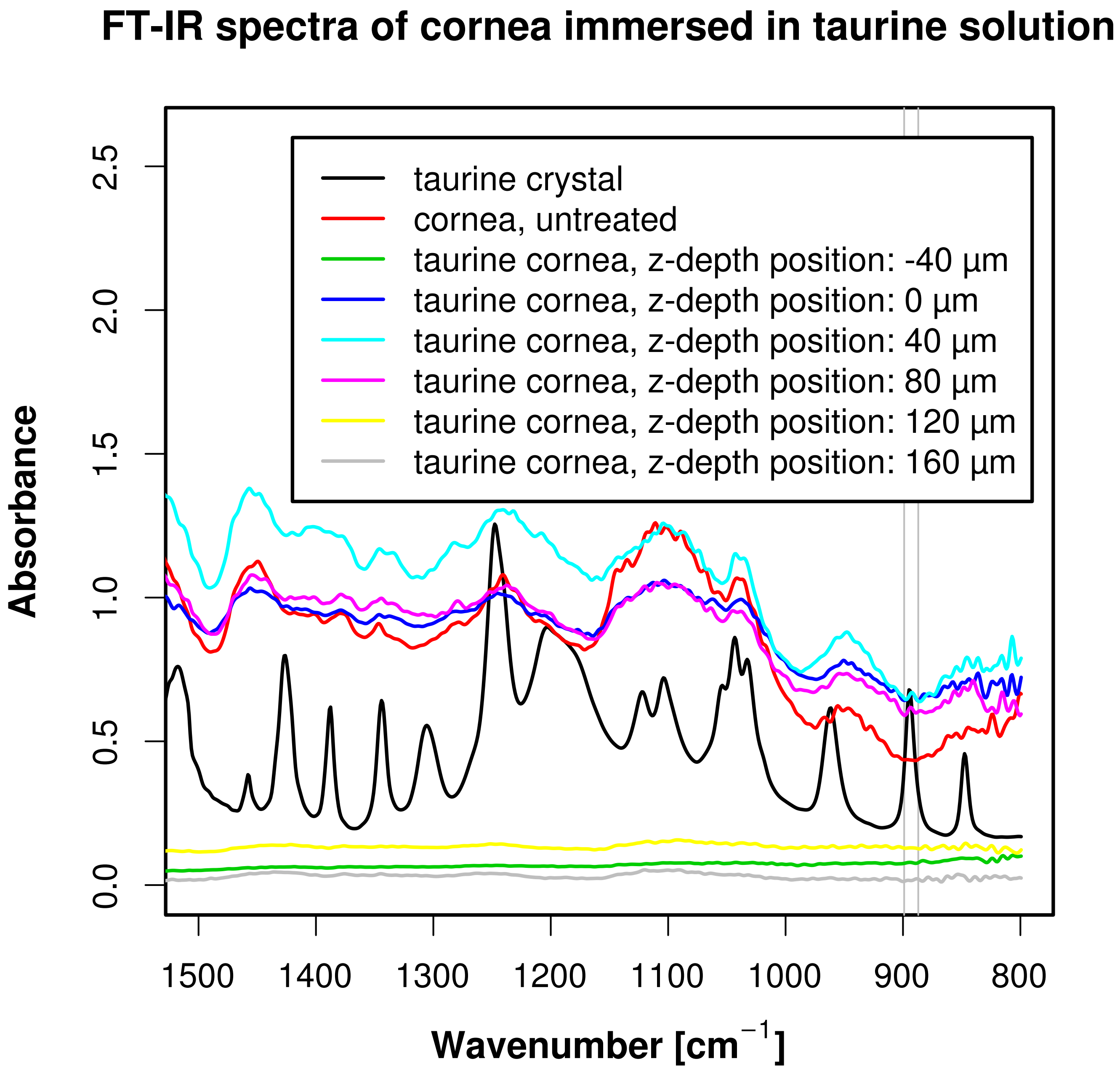

Supplement: Supplementary Information [file srep13868-s1.pdf]
